# A delayed venous repair strategy after penetrating vascular trauma

**DOI:** 10.1016/j.jvscit.2025.101799

**Published:** 2025-04-04

**Authors:** Sahil Patel, Shahabal Khan, Tripti Mathur, Camille Jackson, Shahram Aarabi

**Affiliations:** a Department of Surgery, University of California San Francisco – East Bay, Oakland, CA; b School of Medicine, University of Illinois Chicago, Chicago, IL

**Keywords:** Vascular surgery, Postoperative complication, Delayed venous repair, pulmonary embolism, Trauma surgery

## Abstract

We describe a patient with an external iliac artery and vein injury managed initially with arterial shunting and vein ligation because of hemodynamic instability. The patient underwent a delayed interposition bypass grafts of both external iliac artery and vein. Long-term outcomes were good despite development of postoperative venous thromboembolism. Although it is thought that venous ligation, often completed in a damage-control scenario, precludes venous repair, this case shows a staged approach to venous repair may decrease postoperative morbidity. Further investigation is needed to determine the role of delayed venous repair in improving outcomes.

Iliac vascular injuries occur in approximately 2.3% of abdominal trauma; of these, approximately 8% include combined iliac artery and vein injuries.^1^ A retrospective study of 6262 iliac injuries reported a 30-day mortality rate of 48.7% for combined iliac artery and vein injuries, 16.5% for isolated iliac vein injuries, and 19.3% for isolated iliac artery injuries.^1^ Typically, iliac artery injuries temporized with arterial shunting require arterial reconstruction when the patient is stabilized.^2^ The iliac vein is either shunted, ligated, or repaired, but evidence is mixed for the relative benefits of venous repair.^3^^,^^4^ We present a patient with a combined long-segment external iliac artery and vein injury who underwent delayed iliac venous reconstruction at the time of definitive arterial repair. This patient has agreed to have their case details published.

## Case report

An 18-year-old male with no relevant medical history presented with gunshots to the abdomen and right lower extremity (RLE). He was found to have external iliac artery and vein transections along with small bowel injuries. The common or internal iliac vessels were not injured. Because brisk forward and back bleeding was noted from both ends of the external iliac artery, the artery was considered patent without thrombus. A 10 Fr Argyle shunt was inserted into the artery and secured with 0-silk; patency was confirmed with multiphasic Doppler signal. The iliac vein’s transected ends were ligated for expedience, given the patient’s worsening clinical status. Because of the patient’s prolonged hypotension, vasopressor requirement, and coagulopathy, a temporarily abdominal closure was performed.

Re-exploration was done 6.5 hours after the index operation due to absent Doppler signals in the RLE. A 14 Fr Argyle shunt replaced the previously clotted 10 Fr arterial shunt, and proximal/distal thrombectomies were performed, after which a Doppler signal was found distally. Because RLE swelling was severe, a four-compartment fasciotomy was done. No prophylactic anticoagulation was given after either operation due to bleeding concern.

The next day, the patient returned to the operating room for an abdominal re-exploration, small bowel anastomosis, and fascial closure. Severe RLE edema led us to repair the ligated iliac vein to try to minimize limb morbidity. Heparin was given systemically, and a #6 Fogarty balloon was passed to remove acute clot proximally and distally, until no more return of clot, while the cephalad aspect of the iliac vein was controlled manually to prevent embolization centrally. A cryoartery graft approximately 10 cm long and 8 mm in diameter was used to reconstruct the right external iliac vein in a running end-to-end configuration with 5-0 Prolene in four quadrants. Cryograft was used because no good native vein options were available. Vein patency was confirmed in both directions after Fogarty thrombectomy with brisk bleeding before the anastomosis was completed. For the iliac artery injury, the 14 Fr argyle shunt was removed, a #5 Fogarty balloon passed proximally and distally to remove acute thrombus, and arterial reconstruction was done in a running end-to-end configuration with a reversed contralateral greater saphenous vein graft approximately 10 cm in length with a distended diameter of 5 mm with no concern for size mismatch. A greater saphenous vein graft and cryoartery were used to reconstruct the artery and vein, respectively, because of abdominal contamination from small bowel injuries. At the end of the operation, there were multiphasic Doppler signals in the arterial graft and at the ankle, as well as a venous Doppler signal in the venous repair. The patient tolerated the procedure well, and his RLE edema improved immediately. The patient received prophylactic-dosed Lovenox with a plan for therapeutic dosing when his bleeding risk has minimized. Deep vein thrombosis (DVT) prophylaxis also included ambulation twice daily, leg elevation, and sequential compression device on the left lower extremity.

Four days after arterial and venous repair, the patient’s oxygen saturation dropped. Duplex ultrasound of the RLE showed a noncompressible common femoral vein and a patent reconstructed iliac vein. Subsequent computed tomography angiography showed acute pulmonary emboli (PE) in the lungs bilaterally. Therapeutic heparin was started, which eventually was transitioned to a 6-month course of Xarelto. The patient’s fasciotomy wounds were closed with interrupted 2-0 nylon horizontal mattress sutures 6 days after the index operation. Duplex ultrasounds of the arterial and venous grafts 13 days after arterial/venous repair confirmed patency and distal flow, along with no further evidence of DVT in the common femoral vein. After 2 months, the patient was ambulating independently, had minimal RLE edema, 2+ distal RLE pulses, and no sequalae in cardiopulmonary status.

## Discussion

The ideal approach for venous injuries is still debated. In cases of hemodynamic instability, venous shunting or ligation is often favored, due to their expedience in stabilizing the patient.^5^ Literature on venous ligation is mixed. Some studies show ligation does not significantly increase the risk of muscle debridement, acute kidney injury, secondary interventions, or amputation when compared with repair.^1^^,^^6^ Other studies show venous ligation can lead to chronic venous insufficiency,^3^ as well as higher rates of fasciotomy and secondary amputations than immediate repair.^4^ Isolated iliac vein injuries treated with ligation have been associated with higher mortality rates than those managed with venous repair,^1^ suggesting repair should be performed when feasible.

The Eastern Association for the Surgery of Trauma suggests that venous injuries should be repaired to decrease incidence of edema, shock, and mortality as long as the patient is not hemodynamically unstable and repair will not delay necessary trauma care.^6^, ^7^, ^8^, ^9^ Although pathophysiologic mechanisms underlying improved outcomes from venous repair are not well-understood, venous shunting after vascular injury appears to offer several advantages, including reduced vascular resistance and better venous drainage.^9^ Moreover, immunohistochemical markers (eNOS, HSP70) suggest that venous ligation is associated with more intense ischemic findings than venous shunting.^10^ The improved venous drainage and superior immunohistochemical markers seen with shunting can act as surrogate measures to explain the improved outcomes from venous repair.

The expected benefits from venous repair and our patient’s significant postoperative edema led us to undertake a delayed external iliac vein repair. The results were positive: RLE edema improved and our patient survived beyond 30 days with a functional limb, despite the 48.7% 30-day mortality rate for combined iliac artery and vein injuries.^1^ Our patient did have postoperative DVT/PE. No clear consensus exists about whether venous repair significantly raises the risk of venous thromboembolism (VTE) compared with ligation. Some studies suggest a possible association between venous repair and a higher incidence of VTE,^3^^,^^6^^,^^11^ but others report that venous repair and ligation have similar rates of DVT (8%-15%) and PE (1%-2%).^1^^,^^12^^,^^13^

Case reports have described how delayed venous repair improved distal edema after delayed repair of the inferior vena cava and superior mesenteric vein,^14^, ^15^, ^16^ but the existing literature is heterogenous, encompassing a range of venous injuries and complications, such as concomitant arterial and nonvascular injuries. Our case adds to this complex landscape by showing that a delayed approach to venous reconstruction after ligation in traumatic injuries might improve postoperative limb morbidity. This case also underscores the need for animal trials or clinical studies to understand management of venous injuries.

## Conclusion

In patients with penetrating trauma, although venous repair should be completed if feasible, hemodynamic instability or concerns about delays in hemorrhage control often led to venous ligation. Delayed venous reconstruction can be an alternative if there is vigilance regarding the risk of postoperative VTE.

## Funding

None.

## Disclosures

None.
